# Pathogenomic Analysis of Wheat Yellow Rust Lineages Detects Seasonal Variation and Host Specificity

**DOI:** 10.1093/gbe/evx241

**Published:** 2017-11-21

**Authors:** Vanessa Bueno-Sancho, Antoine Persoons, Amelia Hubbard, Luis Enrique Cabrera-Quio, Clare M Lewis, Pilar Corredor-Moreno, Daniel C E Bunting, Sajid Ali, Soonie Chng, David P Hodson, Ricardo Madariaga Burrows, Rosie Bryson, Jane Thomas, Sarah Holdgate, Diane G O Saunders

**Affiliations:** Crop Genetics, John Innes Centre, Norwich Research Park, United Kingdom; ational Institute of Agricultural Botany, Cambridge, United Kingdom; Earlham Institute, Norwich Research Park, United Kingdom; The Sainsbury Laboratory, Norwich Research Park, United Kingdom; The University of Agriculture, Peshawar, Pakistan; The New Zealand Institute for Plant & Food Research, Lincoln, New Zealand; CIMMYT, Addis Ababa, Ethiopia; National Institute of Agricultural Research, INIA, Chile; BASF SE, Agricultural Centre, Limburgerhof, Germany

**Keywords:** pathogenomics, population genomics, *Puccinia striiformis*, wheat yellow rust, plant pathology

## Abstract

Recent disease outbreaks caused by (re-)emerging plant pathogens have been associated with expansions in pathogen geographic distribution and increased virulence. For example, in the past two decades’ wheat yellow (stripe) rust, *Puccinia striiformis* f. sp. *tritici*, has seen the emergence of new races that are adapted to warmer temperatures, have expanded virulence profiles, and are more aggressive than previous races, leading to wide-scale epidemics. Here, we used field-based genotyping to generate high-resolution data on *P. striiformis* genetics and carried out global population analysis. We also undertook comparative analysis of the 2014 and 2013 UK populations and assessed the temporal dynamics and host specificity of distinct pathogen genotypes. Our analysis revealed that *P. striiformis* lineages recently detected in Europe are extremely diverse and in fact similar to globally dispersed populations. In addition, we identified a considerable shift in the UK *P. striiformis* population structure including the first identification of one infamous race known as Kranich. Next, by establishing the genotype of both the pathogen and host within a single infected field sample, we uncovered evidence for varietal specificity for genetic groups of *P. striiformis*. Finally, we found potential seasonal specificity for certain genotypes of the pathogen with several lineages identified only in samples collected in late spring and into the summer, whereas one lineage was identified throughout the wheat growing season. Our discovery of which wheat varieties are susceptible to which specific *P. striiformis* isolates, and when those isolates are prevalent throughout the year, represents a powerful tool for disease management.

## Introduction

Disease outbreaks caused by (re-)emerging plant pathogens have frequently been associated with expansions in pathogen geographic distribution and increased virulence of known pathogens. One example is the wheat yellow (stripe) rust pathogen, *Puccinia striiformis* f. sp. *tritici* (PST), that is present in all wheat growing areas of the world. The resulting disease can cause high yield losses, even in countries where resistant wheat varieties and/or fungicides are used (Wan et al. 2004; Wellings 2011). In the past two decades, new, more aggressive, *P. striiformis* races adapted to warmer temperatures and showing expanded virulence profiles have caused widespread epidemics (Milus et al. 2009). As a case in point, two highly aggressive *P. striiformis* strains (PstS1 and PstS2) were present at epidemic sites across five continents in 2009, reflecting the most rapid and extensive spread of any important plant pathogen to date (Hovmoller et al. 2010). The emergence of high temperature-tolerant *P. striiformis* races has enabled the disease to proliferate in new geographic regions. As these races continue to evolve in their new habitats, this epidemic will likely continue (Hovmoller et al. 2010).

In Europe, *P. striiformis* has re-emerged as a major constraint on wheat production; races recently arising in Europe have overcome many of the major resistance genes in European germplasm. For instance, in 2011, two new *P. striiformis* races collectively known as Warrior (based on the virulence of one initial variant of this group to the wheat variety Warrior) and Kranich (based on the virulence of one variant to the wheat variety Kranich) emerged as serious threats to wheat production (Hubbard et al. 2015; Hovmoller et al. 2016). The Warrior race was identified in many countries across Europe in the first year of detection, whereas reports of the Kranich race have been largely confined to Denmark, Poland, Sweden, and Finland (Hovmoller et al. 2016). These two emergent races differ from the older European races; they produce unusually high quantities of sexual teliospores, induce more pronounced disease symptoms on wheat varieties harboring long-lasting adult plant resistance, and have higher degrees of genetic diversity (Rodriguez-Algaba et al. 2014; Hubbard et al. 2015; Hovmoller et al. 2016). The high levels of genetic diversity within these races is attributed to the fact they originated from sexually recombining populations in Asia, reaching Europe via long-distance aerial dispersal (Hovmoller et al. 2016). As is common among rust pathogens, *P. striiformis* is heteroecious, requiring two hosts to complete its life cycle; *P. striiformis* undergoes asexual reproduction on wheat and sexual reproduction on *Berberis*, where recombination can lead to the emergence of novel genotypes (Jin et al. 2010; Rodriguez-Algaba et al. 2014). *Puccinia striiformis* populations in Europe are highly clonal, with signs of sexual reproduction limited to Asia (Ali 2016). However, long-distance spore dispersal can rapidly spread novel *P. striiformis* genotypes generated in Asia, with the expansion of the fittest genotype driven in new territories by subsequent asexual reproduction (Brown and Hovmoller 2002). In addition, there is a strong indication that humans contribute to the dispersal of wheat yellow rust. For example, humans initially introduced this pathogen into Australia in 1979 (Wellings 2007). Such factors have escalated the frequency of long-distance transmission for *P. striiformis* and led to increasingly unpredictable changes in its population diversity and dynamics in recent years. However, how diverse environments in new habitats such as host genotypes and ecological conditions influence the population dynamics of *P. striiformis* has yet to be truly explored.

Advances in sequencing technologies provide new opportunities to investigate the complexities influencing genetic variation in plant pathogen populations (Guttman et al. 2014). For instance, genome sequencing helped uncover the role of somatic hybridization and gene gain and loss in driving the emergence of host-specialized isolates of *Blumeria graminis* and *Magnaporthe oryzae*, respectively (Menardo et al. 2016; Yoshida et al. 2016). Genomic studies should be particularly valuable for analyzing rust pathogens, which are frequently obligate biotrophs that cannot be axenically cultured in the laboratory environment. Historically, most population studies of rust pathogens have used polymorphic simple sequence repeat (SSR) loci to differentiate pathogen isolates (e.g., *Puccinia triticina*, Kolmer and Acevedo 2016; *Melampsora larici-populina*, Xhaard et al. 2011; *P. striiformis*, Thach et al. 2016). However, the number of SSR loci required to accurately differentiate a population structure can be quite high (Garke et al. 2012). As an alternative, the genomic revolution has enabled the use of hundreds or thousands of single nucleotide polymorphism (SNP) sites, markedly improving the resolving power of population analysis (Singh et al. 2013). A recent study used SNP markers to develop a new RNA-seq-based genotyping technique termed “field pathogenomics,” and detected a complete shift in the UK *P. striiformis* population in recent years (Hubbard et al. 2015). The field pathogenomics technique is so named because it allows the analysis of pathogen population dynamics directly from the field, providing insights into pathogen biology, population structure, and pathogenesis (Derevnina and Michelmore 2015).

Here, we used the field pathogenomics genotyping technique to examine the European *P. striiformis* population in 2014, and through comparative genomics determined the distribution of the emergent Warrior and Kranich race groups on a global scale. A unique feature of the field pathogenomics technique is the ability to investigate the genotypes of both the pathogen and host within a single *P. striiformis*-infected field sample. We used this feature to identify a potentially direct association between *P. striiformis* genotypes and the particular wheat host varieties they infect in the field. This association we uncovered is likely linked to the distinct virulence profiles of *P. striiformis* isolates in different genetic groups. Furthermore, we found that certain *P. striiformis* genotypes appeared to display seasonal specificity with a number of lineages identified only in the late spring and into the summer and one lineage being identified throughout the growing season. This new knowledge regarding potential varietal and temporal specificity for *P. striiformis* lineages was only possible due to recent advances in high-resolution genotyping techniques. Our discovery of wheat variety susceptibility to specific *P. striiformis* isolates that are prevalent at certain times of the year can have considerable impact on directing future disease management approaches.

## Materials and Methods

### Transcriptome Sequencing of *P. striiformis*-infected Leaf Samples

A total of 246 *P. striiformis*-infected wheat, triticale, and rye leaf samples were collected from December 2013 to August 2014 across 16 European countries and stored in RNAlater solution (Thermo Fisher Scientific, Paisley, United Kingdom) as described previously (Hubbard et al. 2015) (supplementary table S1, Supplementary Material online). Additional current and historical *P. striiformis*-infected samples from Pakistan, Ethiopia, Chile, and New Zealand were also obtained. A subset of 133 of these samples were subjected to RNA extraction using a Qiagen RNeasy Mini Kit according to the manufacturer’s instructions (Qiagen, Manchester, United Kingdom). Quality and quantity of extracted RNA were assessed using an Agilent 2100 Bioanalyzer (Agilent Technologies, United Kingdom) and cDNA libraries prepared using an Illumina TruSeq RNA Sample Preparation Kit (Illumina). Libraries were sequenced on the Illumina HiSeq 2500 machine at the Earlham Institute, United Kingdom. The FASTX-Toolkit (version 0.0.13.2) was used for adapter and barcode trimming and quality filtering. The 101-bp (HiSeq) pair-end reads were aligned to the PST-130 assembly as described previously (Hubbard et al. 2015). SAMtools (version 0.1.19) (Li et al. 2009) was used for *P. striiformis* SNP-calling, considering only sites with a minimum depth of coverage of 20×. SNP sites with allelic frequencies ranging from 0.2 to 0.8 were classified as heterokaryotic sites, and those with other allelic frequencies were classified as homokaryotic sites. The 0.2–0.8 boundaries were selected by calculation of the MAF (Major allelic frequency) for all isolates from 2014, which indicated that the number of positions that fall within the 0.8 and 0.9 boundaries represent just 0.59% of the total number of positions considered. Read frequencies were calculated for biallelic heterokaryotic SNP sites and plotted using ggplot2 in R (Ginestet 2011). SnpEff, version 3.6 (Cingolani et al. 2012) was used to identify homokaryotic and heterokaryotic SNP sites that induced synonymous and nonsynonymous substitutions. Illumina reads from all RNA-seq runs were deposited in the European Nucleotide Archive (ENA; PRJEB15280).

### Genome Sequencing of the UK *P. striiformis* Kranich Isolate

Genomic DNA was extracted from dried urediniospores of *P. striiformis* isolate 14/106 using the CTAB method (Chen et al. 1993). A gDNA library was prepared as described previously (Hubbard et al. 2015) and library quality confirmed before sequencing using the Agilent 2100 Bioanalyzer (Agilent Technologies). The library was sequenced on the Illumina HiSeq 2500 machine at the Earlham Institute, United Kingdom. Data filtering was carried out as described earlier and the 101-bp (HiSeq) pair-end reads aligned to the PST-130 assembly (Cantu et al. 2011) as described previously (Hubbard et al. 2015). SNP-calling and SNP analysis were performed as described earlier. The gDNA data were deposited in the European Nucleotide Archive (ENA; PRJEB15280).

### Phylogenetic Analysis of Historical and Current *P. striiformis* Genetic Groups

A maximum likelihood approach was used for all phylogenetic analyses of *P. striiformis* isolates. To identify and record a nucleotide residue, a minimum of 20× depth of coverage was required for sites that differed from the PST-130 reference, with 2× coverage if they were identical to the reference. These sites were used to generate synthetic gene sets for each isolate using the method described previously (Hubbard et al. 2015). Unrooted maximum likelihood trees were constructed using the third codon position of these genes using RaxML 8.0.20 with 100 replicates using the rapid bootstrap algorithm (Stamatakis 2006). Phylogenetic trees were visualized in MEGA6.06. The results from STRUCTURE analysis for the European population were incorporated into the phylogenetic tree using iTOL (Letunic and Bork 2007).

### Population Structure Analysis of *P. striiformis* Field Isolates

The genetic substructuration of *P. striiformis* isolates was assessed using both model-based Bayesian and nonparametric multivariate clustering methods. The Python program StrAuto, version 3.1 (Chhatre and Emerson 2017) was used to execute the Bayesian model-based approach implemented in STRUCTURE, version 2.3.4 (Pritchard et al. 2000). First, sites that introduced a synonymous change in at least one isolate were listed. The nucleotide at this position was then extracted for all isolates. The “admixture” model was used with three to five replicates of 220,000 Markov Chain Monte Carlo generations for a range of *K* values, where *K* is the number of genetic groups. For each run, the first 110,000 generations were discarded as burn-in before collecting data. To identify *K* values, the average log probability (Ln*P*(*D*)) of each *K* value was calculated. However, as STRUCTURE is sensitive to asexual reproduction (Gao et al. 2007), the subdivision of genetic groups was also analyzed with multivariate analysis using discriminant analyses of principal components (DAPC) implemented in the Adegenet package in the R environment (Jombart et al. 2010), a nonparametric approach used without any predetermined genetic model. Using the synonymous sites extracted for STRUCTURE analysis, the number of maximal genetic groups (Kmax) was assessed based on decreasing Bayesian information criterion (BIC) values, as suggested by Jombart *et al.* (2010).

### Assessing Diversity within and between *P. striiformis* Genetic Groups

The diversity within and between all *P. striiformis* genetic groups was assessed by generating synthetic gene sets for each individual isolate that incorporated all heterokaryotic and homokaryotic SNPs from alignments of each isolate to the PST-130 reference. Synthetic genes were combined for all *P. striiformis* isolates within a genetic group, and used to calculate the degree of nucleotide diversity between isolates within a single genetic group using the EggLib software package, version 2.1.2 (De Mita and Siol 2012). The number of SNPs identified within a genetic group from this set of genes was then used to assess the number of SNPs/kb per genetic group.

*P. striiformis* has intra individual polymorphisms due to differences between the two nuclei caused by its dikaryotic status. To assess the divergence between our genetic groups based only on inter individual polymorphisms, the genotype of each isolate was generated using an in-house Python script by merging both haplotypes into just one sequence without phasing (AA->0; AC/CA->1; AG/GA->2; AT/TA->3; CC->4; CG/GC->5; CT/TC->6; GG->7; GT/TG->8; TT->9; missing data ->X). The genotypic diversity was then calculated corresponding to the interindividual diversity within a genetic group and the genetic distance (*D_A_* and *D_XY_*) of these genetic groups using the Egglib software.

To define total genetic variance attributable to intergroup differences, a total of 36,921 synonymous SNP sites were used in Genepop, version 4.2 (Rousset 2008) to calculate the Wright’s *F*_ST_ statistic. This was carried out for all 2013 and 2014 European isolates combined. To further characterize the different *P. striiformis* genetic groups, 427 genes with at least 80% breadth of coverage for all isolates (representing 407,421 positions with an average of 1,256 polymorphic sites) were selected, and their sequences concatenated using an in-house Python script. The excess of heterozygotes (*F*_IS_) and nucleotide diversity (Π) were calculated for all genetic groups using Egglib version 2.1.2 (De Mita and Siol 2012). The number of SNPs between two genetic groups identified for this set of genes was used to compute the number of intergenetic group SNPs/kb.

### KASP Assays

Primers were designed as described previously (Hubbard et al. 2015), carrying standard FAM or HEX compatible tails (FAM tail: 5′-GAAGGTGACCAAGTTCATGCT-3′; HEX tail: 5′-GAAGGTCGGAGTCAACGGATT-3′), with the SNP at the 3′-end. Assays were carried out as described previously (Hubbard et al. 2015) and data were analyzed manually using Klustercaller software (version 2.22.0.5, LGC).

### Virulence Profiling of *P. striiformis* Isolates

*Puccinia striiformis* isolates were screened for compatibility across a set of wheat cultivars possessing known resistances to *P. striiformis* and a subset of cultivars possessing resistances that have yet to be fully described. Infection assays were undertaken with ten technical replicates on seedlings under controlled environmental conditions. Infection reactions were assessed on the first seedling leaf using a 0–4 scale with infection types 3 and 4 considered to represent a compatible interaction between the host genotype and pathogen isolate. Host resistance genes included in the differential set were *Yr1*, *Yr2*, *Yr3*, *Yr4*, *Yr5*, *Yr6*, *Yr7*, *Yr8*, *Yr9*, *Yr10*, *Yr15*, *Yr17*, *Yr24*, *Yr25*, and *Yr32*, as well as Spaldings Prolific, Robigus, Solstice, Warrior, KWS-Sterling, Claire, Crusoe, Ambition, Vuka, Cadenza, Ambition, and Brigadier.

For adult plants, five isolates were tested using an extended variety set. The varieties consisted of the current UK Agriculture and Horticulture Development Board Recommended List, representing commonly grown UK wheat varieties. Each variety was sown as a 30-cm diameter tussock in two replicates in each of five separate trials. Each trial was inoculated with one of the five selected isolates by transplanting infected seedlings into spreader plots within the trial. The spreader plots subsequently provided naturally dispersed inoculum into the variety plots. The percentage leaf area infected was assessed at weekly intervals once the value for the susceptible control (Robigus) reached 5%. An average of these assessments was then taken to represent disease progression throughout the season. The seedling tests were also extended to include the same variety set used in the adult plant tests under controlled environmental conditions. A subset of differentials from the initial pathotype screening was also included to check consistency between tests. Varieties were tested and assessed as described earlier.

### Identification of Wheat Varieties in *P. striiformis*-Infected Field Samples

From the set of ∼90,000 SNP markers genotyped using the wheat Illumina iSelect array (Wang et al. 2014), 21,505 nonredundant SNP loci were selected, including 1,831 that could be used to differentiate wheat varieties where SNP information was available (genotype data, developed by NIAB within the “WAGTAIL” project, are freely available from NIAB upon request). The wheat varieties of the field samples were confirmed as previously described (Hubbard et al. 2015). First, reads were aligned to sequences flanking the 21,505 SNP markers. The SNP sites were then determined using VarScan (Koboldt et al. 2009), with a minimum base quality of 20, and homozygous SNPs reported when the allele frequency exceeded 0.80. A scoring system was used to determine the most likely wheat variety in a particular sample as described previously (Hubbard et al. 2015). In short, if a *P. striiformis*-infected wheat sample matched the SNP site for a particular variety, the position was scored as 1, and if the site partially matched, the score was 0.5. Otherwise, the position was scored as 0. This analysis was carried out for all SNP markers in a sample, and the cumulative score for all varieties was determined. The final score per variety represents the similarity between the *P. striiformis*-infected sample and a particular variety. The highest score corresponds to the most similar variety across varieties where marker data were available.

## Results

### High-Resolution Genotyping of the European 2014 *P. striiformis* Populations

To characterize the diversity of *P. striiformis* across Europe in the 2014 growing season, we collected 246 *P. striiformis*-infected wheat, triticale, and rye samples from December 2013 to August 2014 from fields across 16 European countries (supplementary table S1, Supplementary Material online). We extracted total RNA from a subset of 115 European *P. striiformis*-infected samples and subjected it to direct RNA-seq analysis using the field pathogenomics method (Hubbard et al. 2015). This method allows the genotypic diversity of *P. striiformis* to be characterized directly at the field level. Samples were selected for RNA-seq analysis to maximize their temporal, varietal, and spatial distribution (supplementary table S1, Supplementary Material online). Following quality filtering, reads were aligned to the PST-130 reference genome (Cantu et al. 2011), with an average of 36.87% (19.73±SD) reads aligned (supplementary table S2, Supplementary Material online). To ensure each sample comprised a single *P. striiformis* genotype without bias in allele-specific expression between the two haploid dikaryotic nuclei, we analyzed the distribution of read counts for biallelic single nucleotide polymorphisms (SNPs), determined from alignment against the PST-130 genome, as described previously (Hubbard et al. 2015). The *P. striiformis* mean of read counts at heterokaryotic positions had a single mode at 0.5 for all samples, indicating that each sample had little bias in allele expression and likely represented predominantly a single genotype (supplementary figs. S1–S3, Supplementary Material online).

### The Recently Emergent European *P. striiformis* Lineages Are Related to Widespread Global Populations

To determine the relationship between the European 2014 *P. striiformis* field isolates and the current and historical *P. striiformis* genetic groups, we carried out population genetic analysis. First, we performed RNA-seq analysis of additional *P. striiformis*-infected wheat samples that were sampled in 2014 across three additional continents to place the European population in a global context (supplementary table S3, Supplementary Material online). In addition, to determine the relationship between the 2014 *P. striiformis* population and older archived isolates, we included RNA-seq and genomic data sets for archived isolates from the United Kingdom and France for comparison when available (supplementary table S3, Supplementary Material online). Finally, we carried out RNA-seq analysis of four historical New Zealand *P. striiformis* isolates (supplementary table S3, Supplementary Material online). Following quality filtering and data trimming, high quality reads were aligned to the PST-130 reference genome (supplementary table S4, Supplementary Material online).

**Fig. 1.**
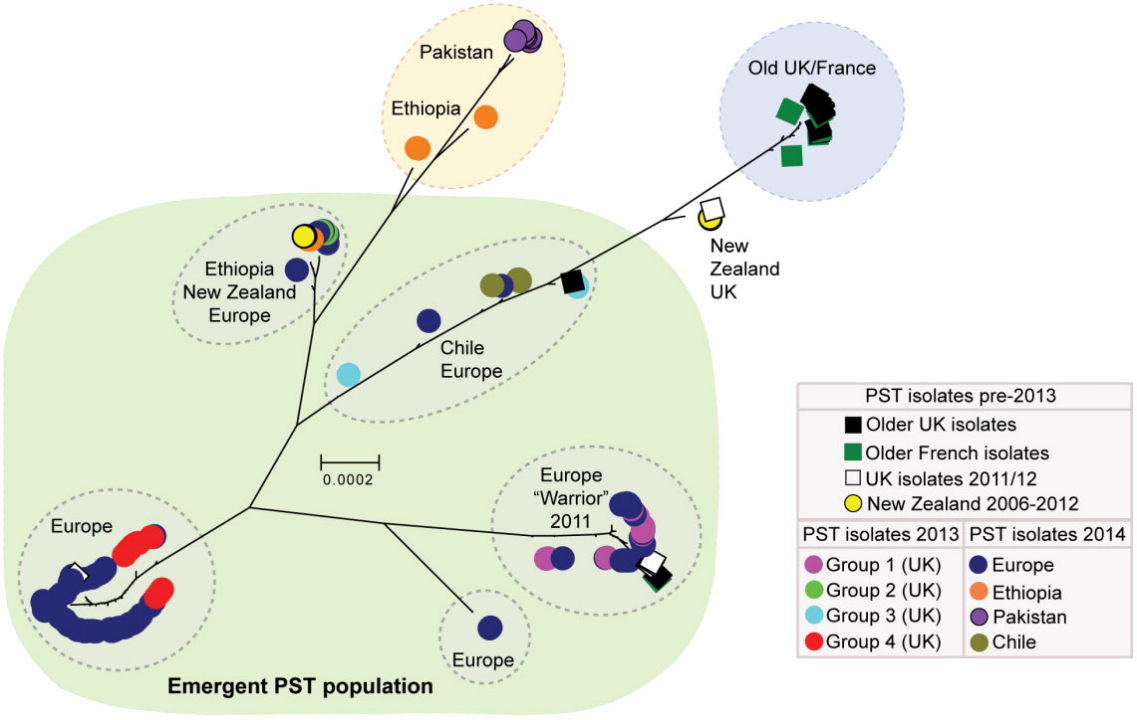
—A number of European *Puccinia striiformis* isolates collected in 2013 and 2014 cluster with isolates from Chile, Ethiopia, and New Zealand. Phylogenetic analysis was carried out on a total of 204 *P. striiformis* isolates using the third codon position of 18,023 PST-130 gene models (5,771,054 sites) and a maximum-likelihood model. Bootstrap values are given in supplementary data S2, Supplementary Material online. Clades are delimitated by dashed ovals; scale bar indicates nucleotide substitutions per site.

**Fig. 2.**
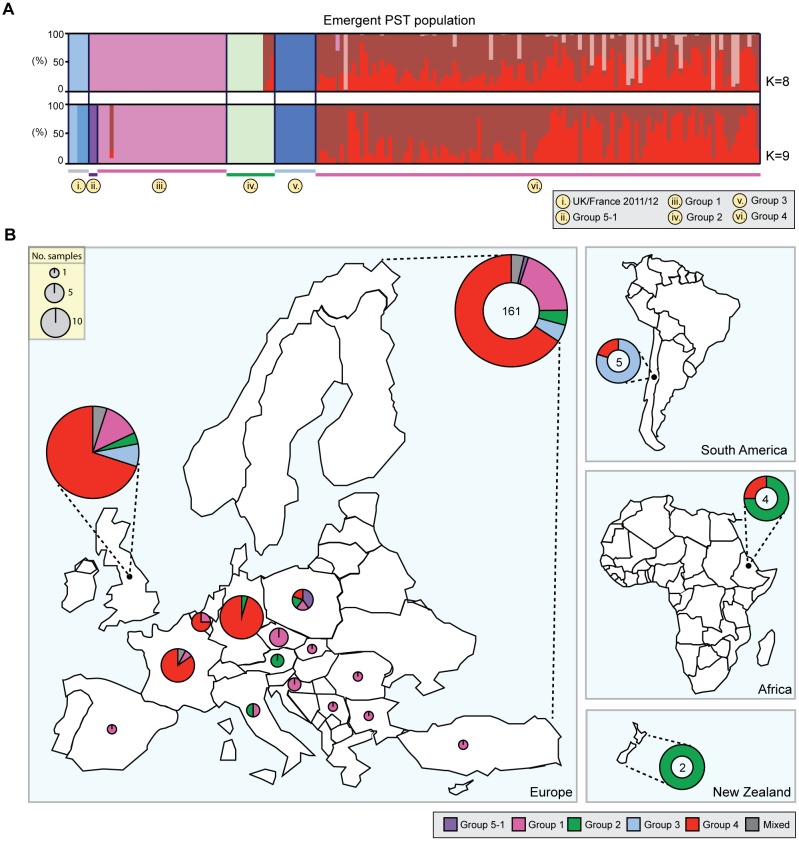
—The emergent European *Puccinia striiformis* lineages were detected on a global scale. (*A*) A list of 70,712 synonymous SNP sites of which 70,565 were biallelic were used to define subdivisions within the population using multivariate discriminant analysis of principal components (DAPC) of the 172 *P. striiformis* isolates belonging to the “Emergent PST population.” The optimal predicted number of population clusters *K* for this data set was eight to nine, giving rise to six clearly definable genetic groups that consisted of 1) the four genetic groups that were previously identified in the United Kingdom in 2013 (Groups iii, iv, v, and vi); 2) a group containing *P. striiformis* isolates from the United Kingdom sourced in 2011 and 2012 (Group i); and 3) an additional subgroup of Group 1, which gave rise to Group 5-1 (Group ii). Bars represent estimated membership fractions for each individual. (*B*) The population clusters identified in the “Emergent PST population” were identified across four geographic regions: Europe, Australasia, South America, and Africa. Numbers indicate total number of samples analyzed from that region.

We then carried out phylogenetic analysis of all 204 *P. striiformis* isolates using the third codon position of 18,023 PST-130 gene models (5,771,054 sites) via a maximum-likelihood model (supplementary data S1 and S2, Supplementary Material online). We found that the 2014 European *P. striiformis* isolates were genetically related to the emergent *P. striiformis* populations that were previously identified in the United Kingdom in 2013 (Hubbard et al. 2015). Additionally, this emergent *P. striiformis* population, which until now had been described only in Europe and at its putative origin, in the center of diversity in the near-Himalayan region of Asia (Hovmoller et al. 2016), was also genetically similar to isolates identified across Australasia, South America, and Africa (fig. 1). To further investigate the genetic structuration among these isolates, we selected 172 *P. striiformis* isolates representing isolates from Europe (collected in 2011–2014), Australasia (2006–2012), South America (2014), and Africa (2014) that were classified as belonging to the emergent *P. striiformis* population based on phylogenetic analysis (fig. 1; “Emergent PST population”). We then generated a list of 70,712 synonymous SNP sites, 70,565 of which were biallelic, and used multivariate discriminant analysis of principal components (DAPC) to define subdivisions within the population. Analysis of the Bayesian Information Criterion (BIC) indicated eight to nine as the optimum clustering solution (supplementary figs. S4 and S5, Supplementary Material online), with isolates clearly assigned to six definable homogeneous groups of individuals (fig. 2*A*). These genetic groups consisted of 1) the four genetic groups that were previously identified in the United Kingdom in 2013, 2) a group containing *P. striiformis* isolates from the United Kingdom and France sourced in 2011 and 2012, and 3) an additional subgroup of Group 1, which gave rise to Group 5-1. We also sought to confirm the number of groups independently using the average log probability (Ln*P*(*D*)) of each *K* value in the model-based clustering program STRUCTURE. The average log probability stabilized at *K* = 6 (supplementary fig. S6*A*, Supplementary Material online); however, no isolates were assigned to three of the genetic groups (supplementary fig. S6*B*, Supplementary Material online). Nonetheless, by considering *P. striiformis* isolates that were previously assigned to the four clearly defined genetic groups identified in 2013 and the phylogenetic analysis, we concluded that the STRUCTURE analysis supported division of the emergent *P. striiformis* population into four homogeneous groups of individuals (supplementary fig. S6*B*, Supplementary Material online). The discrepancies in the results between the two analyses are likely due to assumptions incorporated into the later model-based analysis regarding the underlying population genetics model, particularly Hardy–Weinberg equilibrium or linkage equilibrium, which are ill-suited to an asexually reproducing population (Jombart et al. 2010). Overall, these analyses indicated that the emergent *P. striiformis* population identified initially in Europe is in fact similar to isolates identified on a global scale (fig. 2*B*) and consists of a number of distinct lineages.

### A Shift in the Overall UK *P. striiformis* Population between 2013 and 2014

**Fig. 3.**
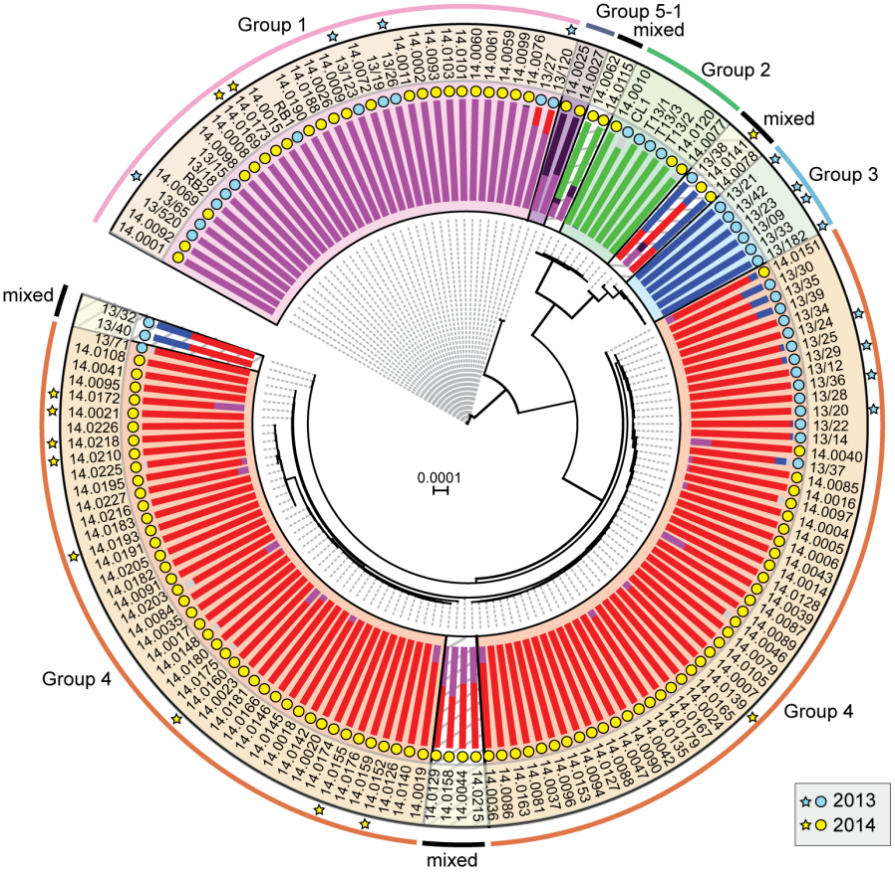
—A shift in the European *Puccinia striiformis* population between 2013 and 2014. Phylogenetic analysis was carried out on thirty-nine 2013 and one hundred and fifteen 2014 *P. striiformis* isolates using the third codon position of 18,023 PST-130 gene models (4,041,039 sites) and a maximum-likelihood model. Bayesian-based clustering of 36,921 biallellic synonymous SNP sites classified the *P. striiformis* field isolates into five distinct population clusters, with Group 5-1 first identified in 2014. Bar charts represent STRUCTURE analysis with each bar representing estimated membership fractions for each individual. Stars indicate isolates purified for virulence profiling in 2013 (blue) and 2014 (yellow). Colored circles represent the year in which the samples were collected. Bootstrap values are given in supplementary data S4, Supplementary Material online. Isolate names are shown in outer ring; scale bar indicates nucleotide substitutions per site.

**Fig. 4.**
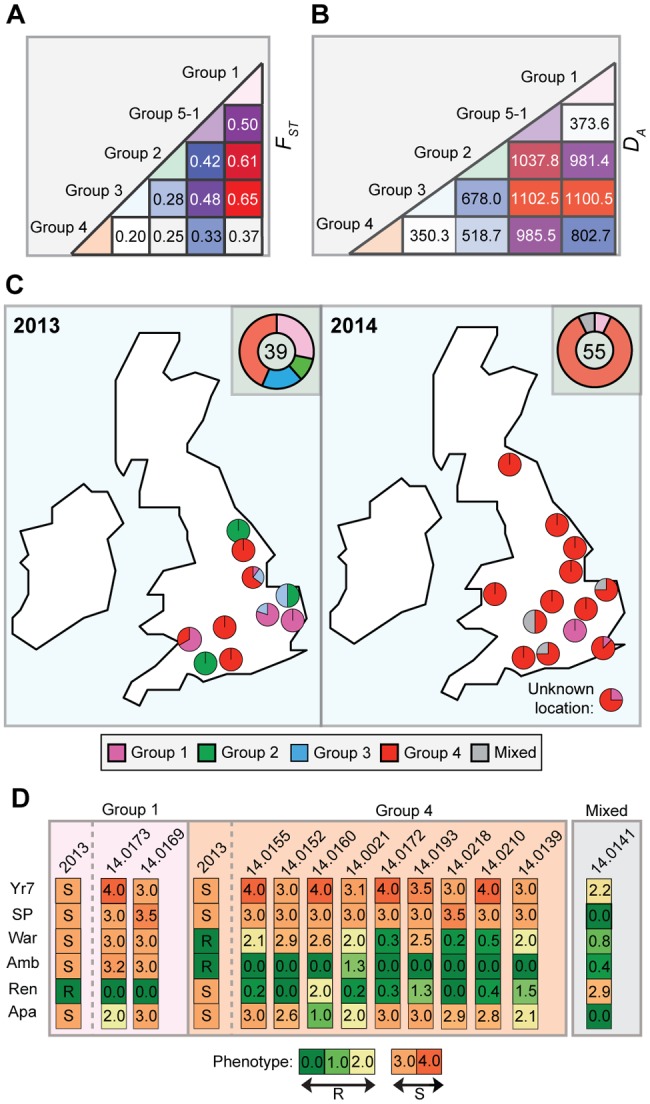
—*Puccinia striiformis* isolates assigned to genetic Group 4 were the most prevalent in the United Kingdom in 2014. (*A*) Pair-wise comparisons between the five European *P. striiformis* population groups indicated that Groups 3 and 4 were most closely related (*F*_ST_ 0.20), while Groups 3 and 1 were the most distantly related (*F*_ST_ 0.65). (*B*) Analysis of the genetic distance (*D_A_*) between groups illustrated that Groups 3 and 4 and Groups 1 and 5-1 had the smallest genetic distance (350.3 and 373.6, respectively). (*C*) Group 4 *P. striiformis* isolates dominated in the United Kingdom and were widespread in 2014, while Groups 2 and 3, which were prevalent in 2013, were absent among the 2014 UK *P. striiformis* isolates analyzed. Pie charts indicates prevalence of *P. striiformis* genetic groups in each county sampled; doughnut chart shows cumulative prevalence of genetic groups for the United Kingdom per year with numbers indicating the total number of isolates analyzed per year. (*D*) Virulence profiles for the genetic groups were largely conserved between isolates belonging to the same group. Two *P. striiformis* isolates from Group 1 and nine from Group 4 were inoculated onto a series of 46 differential wheat varieties; the six discriminative results are shown: Yr7, AvocetS-Yr7 near isogenic line; Sp, Spaldings Prolific; War, Warrior; Amb, Ambition; Ren, Rendezvous; Ap, Apache. Disease severity was recorded 16- to 20-day post inoculation. Scale: 0 (green) to 4 (red), with 0–2 being resistant (R) and 3–4 being susceptible (S).

In 2013, we identified four distinct emergent lineages within the UK population (Hubbard et al. 2015). To determine the prevalence of these lineages across Europe in 2014, we carried out a comparative analysis with the 2013 data set (Hubbard et al. 2015). First, we undertook a phylogenetic analysis of 39 *P. striiformis* isolates from 2013 and 115 from 2014 using the third codon position of 18,023 PST-130 gene models (4,041,039 sites) via a maximum-likelihood model (fig. 3; supplementary data S3 and S4, Supplementary Material online). Next, we generated a list of 36,921 biallelic synonymous SNP sites. We used the average log probability (Ln*P*(*D*)) in the program STRUCTURE, which supported the division of the combined 2013 and 2014 *P. striiformis* isolates into five genetic groups (fig. 3 and supplementary fig. S7, Supplementary Material online). We confirmed the number of groups independently using DAPC analysis, which also discriminated five genetic groups (supplementary fig. S8, Supplementary Material online). One lineage was only found in the 2014 *P. striiformis* genetic group (Group 5-1). Pair-wise comparisons of the five genetic groups generated a range of *F*_ST_ values, with Groups 3 and 1 being the most distantly related (*F*_ST_ 0.65) and Groups 3 and 4 being the most closely related (*F*_ST_ 0.20) (fig. 4*A*). To determine the level of asexuality in the various genetic groups, we calculated the level of intrapopulation differentiation using the excess of heterozygotes *F*_IS_ and linkage disequilibrium of intrapopulations using rbarD. This analysis tended to confirm the asexuality in the European *P. striiformis* population, with Groups 1, 2, 3, and 4 having a very negative *F*_IS_ (−0.65, −0.62, −0.64, and −0.67, respectively; table 1). All four genetic groups had positive rbarD values (table 1), which indicates an association between alleles, thereby confirming clonality. We then calculated the number of SNPs/kb within and between genetic groups (supplementary table S5, Supplementary Material online). As expected the average number of SNPs/kb was higher between genetic groups (3.1) than within (2.14). Interestingly, Group 5-1 had the highest *F*_IS_ value (−0.44), indicating a smaller excess in heterozygosity than the other genetic groups, with the smallest number of SNPS/kb (0.79, supplementary table S5, Supplementary Material online). This group was newly identified in the United Kingdom in 2014 and together these results could indicate a bottleneck leading to a decrease of diversity following the emergence of this genetic group.

**Table 1:** Asexuality in the European *Puccinia striiformis* Population Was Confirmed Using the Inbreeding Coefficient *F*_IS_ and Linkage Disequilibrium Analysis Using rbarD

Genetic Group	Genotypic and Nucleotide Diversity		rbarD and *F*_IS_		
	Genotype	Nucleotide	No. of Genes	rbarD	*F*_IS_
1	1.34×10^−04^	3.59×10^−4^	2,634	1.51×10^−2^	−0.65
2	4.54×10^−4^	1.48×10^−3^	6,143	2.01×10^−2^	−0.62
3	2.03×10^−4^	1.34×10^−3^	8,045	9.86×10^−3^	−0.64
4	4.21×10^−4^	1.32×10^−3^	462	1.55×10^−2^	−0.67
5-1	5.65×10^−5^	5.21×10^−4^	6,375	N.D.	−0.44
Average	2.54×10^−4^	1.00×10^−3^	4,731.8	1.51×10^−2^	−0.60
SD	1.76×10^−4^	5.21×10^−4^	3,094.4	4.2×10^−3^	0.10

Due to the recent identification of Group 5-1, we calculated the net pairwise genetic distance between genetic groups (*D_A_* distance; Nei et al. 1983) and the pairwise distance (*D_XY_*; Nei and Kumar 2000) using the genotypes of these isolates to avoid intraindividual diversity. The largest genetic distance was identified for the pairwise comparisons between Groups 1 and 3, Groups 5-1 and 2, and Groups 5-1 and 3 (*D_A_*: 1,100.5, 1,037.8, and 1,102.5, respectively; *D_XY_*: 1,158.9, 1,132.7, and 1,149, respectively), with an average *D_A_* of 793.1 (295.0±SD) and *D_XY_* of 885.3 (286.7±SD) for all pairwise comparisons (fig. 4*B* and supplementary table S6, Supplementary Material online). The smallest genetic distance was found for the pairwise comparisons between Groups 3 and 4 (*D_A_*: 350.3; *D_XY_* 462) and between Groups 1 and 5-1 (*D_A_*: 373.6; *D_XY_*: 407.7; fig. 4*B* and supplementary table S6, Supplementary Material online). The particularly low *D_A_* and *D_XY_* distances displayed for Groups 5-1 and 1 and Groups 3 and 4 could indicate the recent divergence of each pair of groups from a recent common ancestor. Alternatively, diversification within one genetic group might have led to the genetic differentiation of the second group. Next, we calculated the genotypic diversity of these genetic groups (π) and compared it with the nucleotide diversity calculations (table 1). Interestingly, Group 5-1 displayed very low genotypic diversity compared with nucleotide diversity (table 1), indicating that this group might have undergone a founder event.

Finally, we investigated the prevalence of these five genetic groups of *P. striiformis* specifically in the United Kingdom in 2014 and compared the results directly with the 2013 UK-specific data. Groups 2 and 3 were absent among the UK samples selected in 2014 (fig. 4*C*). However, *P. striiformis* isolates belonging to Group 2 were previously only identified on triticale, and no triticale samples from the United Kingdom were analyzed in 2014. Furthermore, we noted a significant enrichment (χ^2^, *P* < 0.005) of *P. striiformis* isolates in Group 4 when comparing isolates between 2013 and 2014. These findings suggest that Group 3 may have (at least in part) been displaced in 2014 in the United Kingdom and that Group 4 isolates increased in prevalence (44% in 2013 vs. 84% in 2014) and diversity during this time (nucleotide diversity 1.25×10^−3^ 2013, 1.39×10^−3^ 2014; table 2).

**Table 2:** **Nucleotide Diversity for 2013 and 2014 *Puccinia striiformis* Isolates Belonging to the Four Emergent Genetic Groups in** the **United Kingdom**

	Genetic Group	SD	Nucleotide Diversity	Number of Genes
2013	1	1.12×10^−3^	4.76×10^−4^	5,883
	2	1.87×10^−3^	1.45×10^−3^	6,550
	3	1.71×10^−3^	1.35×10^−3^	8,045
	4	1.56×10^−3^	1.25×10^−3^	6,797
2014	1	1.33×10^−3^	4. 78×10^−4^	4,543
	4	1.75×10^−3^	1.39×10^−3^	467

### Virulence Profiling Reveals the Presence of the Kranich Race in the United Kingdom in 2014

To determine how the observed population structure in the 2014 *P. striiformis* population was reflected in the phenotypic characteristics of the *P. striiformis* field isolates, we purified and cultured a subset of isolates for virulence profiling. Two *P. striiformis* isolates from Group 1, nine from Group 4, and one isolate that could not be clearly assigned to any genetic group (“mixed”) were inoculated on a series of 46 differential wheat varieties. Disease severity was recorded in seedling tests at 16- to 20-day postinoculation (fig. 4*D* and supplementary table S7, Supplementary Material online). Consistent with our previous analysis (Hubbard et al. 2015), we identified a direct link between the genotype and pathotype of the genetically distinct lineages in the UK *P. striiformis* population. However, *P. striiformis* isolates belonging to Group 4 appeared to have lost virulence to Rendezvous in 2014 compared with isolates within the same genetic group from 2013. This finding suggests that Group 4 isolates may have diverged between 2013 and 2014.

**Fig. 5.**
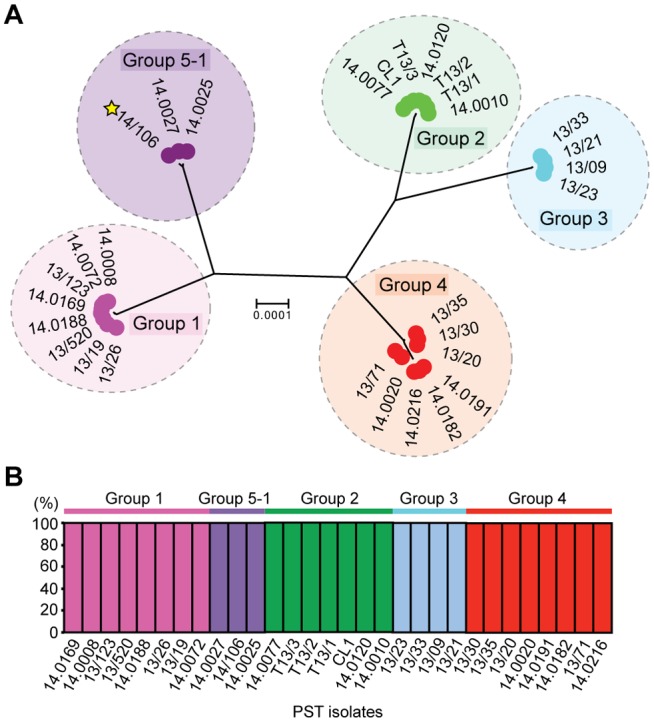
—The UK *Puccinia striiformis* Kranich race isolate belongs to genetic Group 5-1. (*A*) The UK *P. striiformis* Kranich race isolate 14/106 was genetically similar to two isolates from Poland. Phylogenetic analysis was carried out with the UK *P. striiformis* isolate 14/106 and representative isolates from each of the genetic groups identified in the 2013 and 2014 data sets. We used the third codon position of 18,023 PST-130 gene models (5,636,425 sites) and a maximum-likelihood model for the phylogenetic analysis. Genetic groups are delimitated by dashed ovals; scale bar indicates nucleotide substitutions per site. (*B*) Multivariate analysis using discriminant analyses of principal components (DAPC) assigned the UK *P. striiformis* Kranich isolate to genetic Group 5-1. A total of 29 representative isolates were selected for analysis with isolate 14/106, which represented an average of five isolates from each of the five genetic groups. DAPC analysis was undertaken using 33,431 biallelic synonymous SNP sites. Bars represent estimated membership fractions for each individual.

In addition, since we selected only a subset of the UK *P. striiformis* isolates that were subjected to phenotypic analysis for complementary genotypic analysis, we also explored the virulence profiles of the full set of 29 UK *P. striiformis* isolates from 2014 in seedling tests and investigated five of these more comprehensively using a wider set of varieties (supplementary table S7, Supplementary Material online). We identified a notable *P. striiformis* isolate (14/106) with virulence on a broader set of varieties at both the seedling and adult plant stages (supplementary tables S7 and S8, Supplementary Material online), which may represent the emergence of a new *P. striiformis* race in the United Kingdom in 2014. Subsequent independent evaluation of this isolate indicated that the virulence profile was consistent with that of the recently reported Kranich race (Hovmoller et al. 2016) (Hovmoller M [GRRC], personal communication), including avirulence to *Yr4* and the variety Spaldings Prolific and virulence for *Yr8* (supplementary table S7, Supplementary Material online). To investigate this *P. striiformis* isolate further and to compare it with the genetic groups we defined in the European *P. striiformis* population, we carried out full genome resequencing of isolate 14/106. Following alignment to the PST-130 reference genome, we performed phylogenetic analysis of *P. striiformis* isolate 14/106 and representative isolates from each of the genetic groups identified in the 2013 and 2014 data sets. We used the third codon position of 18,023 PST-130 gene models (5,636,425 sites) and a maximum-likelihood model for the phylogenetic analysis (fig. 5*A*).

We then generated a list of 33,482 synonymous SNP sites, 33,431 of which were biallelic, and used both Bayesian clustering in the program STRUCTURE and DAPC analysis to assign the *P. striiformis* isolate (14/106) to a particular genetic group within the European data set. A total of 29 representative isolates from 2013 and 2014 were selected for analysis of *P. striiformis* 14/106, which represented an average of five isolates from each of the five genetic groups identified previously (fig. 3). Analysis using the program STRUCTURE identified only four distinct genetic groups, as indicated by the average log probability (Ln*P*(*D*)) (supplementary fig. S9, Supplementary Material online). However, DAPC analysis resolved all five genetic groups (supplementary fig. S10, Supplementary Material online). The lower number of genetic groups identified using STRUCTURE was likely due to the lower sample number, which caused isolates from genetic Group 3 to be clustered with the relatively closely related Group 4 isolates (*F*_ST_ 0.20; fig. 4*A*). However, in both instances, we showed that *P. striiformis* isolate 14/106 was grouped with two isolates identified in Poland belonging to genetic Group 5-1 (fig. 5). This supports the assignment of Group 5-1 as the Kranich race and is the first, to our knowledge, report of a Kranich race incursion in the United Kingdom.

### *Puccinia striiformis* Genotypes Display Seasonal and Varietal Specificity

**Fig. 6.**
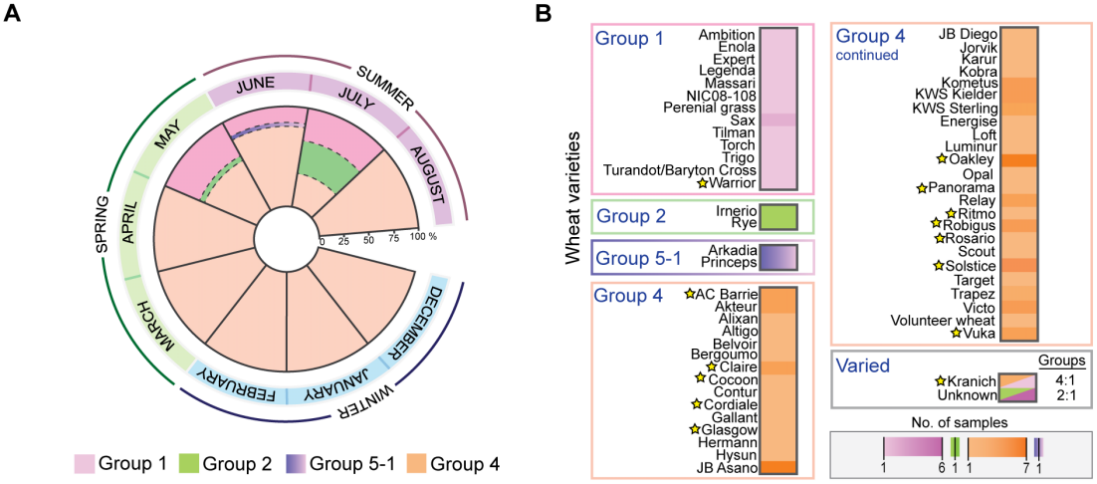
—Particular *Puccinia striiformis* genotypes display seasonal and varietal specificity. (*A*) *Puccinia striiformis* isolates belonging to Groups 1, 2, and 5-1 were only identified in samples collected in May, June, and July. In contrast, isolates from Group 4 were identified throughout the growing season. (*B*) A total of 98% of the 56 wheat varieties analyzed harbored *P. striiformis* isolates from a single genetic group. Stars indicate wheat varieties that were confirmed using single nucleotide polymorphism marker analysis.

In the 2014 growing season, we collected *P. striiformis*-infected samples from December 2013 to August 2014. Isolates belonging to Groups 1, 2 and 5-1 were identified only in samples collected in May, June, and July (fig. 6*A*), whereas *P. striiformis* isolates from Group 4 were identified throughout the growing season (fig. 6*A*). This result was independent of the geographic locations of the samples; for instance, ∼50% of samples from 2014 were collected from various locations across the United Kingdom. To further investigate the apparent seasonal specificity for certain genotypes of *P. striiformis* and rule out yearly bias, we generated a PCR-based assay using SNPs specific to each genetic group to rapidly screen *P. striiformis*-infected wheat samples collected across the United Kingdom in 2015. A total of 42 samples were genotyped from December 2014 to July 2015 and as noted in the 2014 field season, *P. striiformis* isolates from Group 1 were only identified late in the spring and into the summer, whereas isolates from Group 4 were identified throughout the season (supplementary table S9 and fig. S11, Supplementary Material online). No isolates from Groups 5-1 and 2 were identified in the sample set from 2015. We speculate that the ability of *P. striiformis* isolates in Group 4 to infect wheat throughout the growing season may have (in part) led to their predominance in the United Kingdom.

Next, we investigated whether particular *P. striiformis* genotypes showed any preference for wheat varieties that they infected in the field. Where possible, we used our data sets and a set of 1,831 differential wheat SNP markers to confirm the wheat variety in a particular *P. striiformis*-infected sample, as described previously (Hubbard et al. 2015). Thirty-eight *P. striiformis*-infected samples were collected from wheat varieties with available SNP markers and thereby could be used to confirm the wheat variety in each sample. Of the 38 *P. striiformis*-infected wheat samples, we could confirm the variety for 32 (84.21%), where in multiple cases the variety was distributed across different geographic regions (supplementary figs. S12–S14, Supplementary Material online). Next, we assessed all 108 *P. striiformis*-infected samples for which the wheat variety was reported and (where possible) confirmed. We found that 98% of the 56 wheat varieties harbored *P. striiformis* isolates from a single genetic group. Only the Kranich variety harbored genetically distinct *P. striiformis* isolates (Group 1 and 4; *F*_ST_ 0.37; figs. 4 and 6*B*). Therefore, we detected a direct association between *P. striiformis* genotypes and the wheat host pedigrees they infected, which is likely linked to the distinct virulence profiles between isolates in different genetic groups.

## Discussion

### High-Resolution Genotyping Reveals the Emergent European *P. striiformis* Lineages Are in Fact Part of Globally Distributed Populations

Recent advances in genomic-based sequencing technologies have provided opportunities to explore plant pathogen populations at an unprecedented resolution. For instance, such advances in molecular epidemiology can be exploited to rapidly identify the source(s) of harmful genotypes (Lyon et al. 2016), disease reservoirs (Vanhove 2016), origin and lines of descent (McCann et al. 2013), and population structure and its relationship with the emergence of new virulent genotypes (Gladieux et al. 2015). Here, we used RNA-seq-based field pathogenomics to investigate wheat yellow rust population dynamics directly in the field, exploring the diversity of *P. striiformis* within Europe and beyond. This topic is of particular interest given the complete shift in the yellow rust population reported in Europe in recent years, with the emergence of a number of new lineages, including the infamous “Warrior” and “Kranich” races (Hubbard et al. 2015; Hovmoller et al. 2016). In this study, we compared these European *P. striiformis* lineages with isolates from Ethiopia, Chile, New Zealand, and Pakistan. These emergent European *P. striiformis* lineages likely originated from the potential center of diversity in the near-Himalayan region (Hovmoller et al. 2016). This finding, combined with the current data, suggest that this newly emergent *P. striiformis* population, which had only previously been reported at its putative origin in Asia and in Europe, is in fact similar to isolates identified on a global scale, across at least five out of seven continents (Europe, Asia, Africa, South America, and Australasia). However, a previous analysis of isolates collected pre-2008 from East Africa and South America showed that these areas harbored races that were genetically different to the European lineages at that time (Hovmoller et al. 2016). Therefore, our study indicates that the incursion of the emergent *P. striiformis* lineages into Ethiopia and Chile is likely relatively recent. This favors the notion that long-distance dispersal can rapidly spread novel *P. striiformis* genotypes into new territories on a global scale.

### Unprecedented Diversity in the European *P. striiformis* Population

Traditionally, the pre-2011 European yellow rust population propagated asexually, with mutation driving race variability within the confines of Europe, thereby leading to a single clonal genetic group (Hovmoller and Justesen 2007). However, one feature of the emergent *P. striiformis* populations in Europe is the unusual scale of inter- and intradiversity (Hubbard et al. 2015). Furthermore, our comparative analysis of the UK genetic groups between 2013 and 2014 revealed a potential shift in the genetic diversity of the *P. striiformis* population, with considerable diversification within one genetic group, Group 4. This could be due to 1) rapid diversification and expansion of Group 4 between 2013 and 2014 in the United Kingdom, 2) a new exotic incursion of *P. striiformis* isolates with a similar genetic background and/or 3) the small sample size in 2013 not being truly representative of the existing *P. striiformis* diversity. Regardless, such high levels of diversity within Group 4 may reflect differences in life history strategies of the new *P. striiformis* lineages, such as an increase in the rate of somatic hybridization, which would enhance genetic variability.

For rust pathogens, intraspecific and interspecific hybridization can contribute to genetic diversity (Park and Wellings 2012). In addition, somatic hybridization is more likely to occur between allopatric pathogen lineages or species that are brought together from geographically separated epidemiological areas (Stukenbrock and McDonald 2008). The high level of genetic differentiation between the different emergent *P. striiformis* lineages identified in the United Kingdom (*F*_ST_ range 0.20–0.65) is consistent with the notion that the *P. striiformis* population recently arose from multiple exotic incursions from distinct geographical sources (Hubbard et al. 2015). The loss of isolation by distance, which may have previously maintained reproductive isolation, could then lead to more frequent outcrossing between these emergent lineages (Stukenbrock and McDonald 2008). However, the role of hybridization in contributing to the unprecedented wide-scale diversification of certain emergent *P. striiformis* lineages remains to be established. In addition, the low *D_A_* and *D_XY_* distance between Groups 1 and 5-1 is consistent with the idea that either the divergence of both groups from a recent common ancestor or diversification within Group 1 led to the genetic differentiation of Group 5-1. This could indicate a potential bottleneck leading to a decrease of diversity following the emergence of this particular genetic group.

### Precision Pathogen Surveillance as a Powerful Disease Management Tool

High-resolution pathogen genotyping allows pathogen populations to be rapidly defined and tracked in exceptional detail. For instance, the field pathogenomics genotyping technique was recently employed in the rapid response to the first severe outbreak of wheat blast in Asia. Using this approach, the causal agent was confirmed to be a wheat-infecting lineage of *Magnaporthe oryzae*, and the origin was determined to be South America, within just a few weeks of sample collection in the field (Islam et al. 2016). For *P. striiformis*, the ability to accurately genotype both the pathogen and host directly in the field using this technique enabled us to study the coexistence of *P. striiformis* genetic groups and their hosts. The coexistence of distinct genetic groups of a plant pathogen on the same host is widespread (e.g., *Melampsora larici-populina*, Persoons et al. 2016; *Botrytis cinerea*, Fournier and Giraud 2007; *Puccinia striiformis* f. sp. *tritici*, Hubbard et al. 2015).

The niche theory implies three methods of coexistence: 1) space partitioning, 2) time partitioning, and 3) resource partitioning (Amarasekare 2003). Of these three, time partitioning has to date been less well studied. However, evolutionary invasion analysis did reveal that divergence of plant pathogens can be due to time partitioning based on periodic host absence (Hamelin et al. 2011). Seasonality can also play an important role in the management of host–pathogen interactions based on contact rates, variation in encounters with infective stages in the environment and changes in the host immune system (Altizer 2006). In this study, we determined that *P. striiformis* may use both resource and time partitioning. For instance, we provide evidence that specific genetic groups of *P. striiformis* display a high degree of wheat varietal specificity in the agro-ecosystem, allowing the coexistence of specialized genetic groups of *P. striiformis* on the same host species. Furthermore, we also identified a degree of time partitioning for *P. striiformis* isolates in Groups 1, 2, and 5-1 that were only identified in late spring and summer compared with isolates in Group 4 that were present throughout the growing season from December through to August. This may have in part contributed to the increased prevalence of Group 4 isolates in the United Kingdom between 2013 and 2014. These observations could suggest an association between certain pathogen genotypes and warmer environmental conditions later in the season. In Southern and Northern France in the 1980s and 1990s, *P. striiformis* isolates were recovered that displayed clear temperature-specific adaptation (Mboup et al. 2012). Alternatively, these observations could reflect more effective resistance-mediated control of Group 4 isolates by adult plants late in the season, thereby restricting Group 4 prevalence and enabling Groups 1 and 2 isolates to thrive. These possibilities should be explored in future studies.

## Conclusions

As global trade continues to expand and people increasingly vacation in more remote locations, the spread of pathogens into new territories is inevitable. For wheat yellow rust, we have already witnessed the devastation caused by the human-assisted introduction of *P. striiformis* into Australia (Brown 1984). Therefore, there is an urgent need for robust, rapid, high-resolution genotyping techniques to track and define a pathogen population’s structure and dispersal on a global scale. Here, we leveraged recent advances in field-based genotyping to generate high-resolution data on *P. striiformis* genetics. This analysis revealed that races of *P. striiformis* that recently emerged in Europe are in fact similar to populations identified on a global scale. Furthermore, we uncovered the possibility of seasonal and varietal specificity in genetic groups of *P. striiformis*. Knowing which wheat varieties are susceptible to specific *P. striiformis* isolates prevalent at certain times of the year could help agronomists fine-tune disease management strategies accordingly. The scale and frequency of emerging diseases will continue to increase, making the integration of high-resolution genotyping into international surveillance programs important as emergent plant pathogens become increasingly unpredictable. 

## Supplementary Material

Supplementary data are available at *Genome Biology and Evolution* online.

## Supplementary Material

Supplementary Informations
