# Supplementary material for: Pathogenomic Analysis of Wheat Yellow Rust Lineages Detects Seasonal Variation and Host Specificity
Source: Genome Biol Evol. 2017 Nov 21;9(12):3282–96. doi: 10.1093/gbe/evx241 (PMC5730935; doi:10.1093/gbe/evx241)
Supplement: Supplementary Informations [file evx241_supp.zip › Supporting Information - Figures.pdf]

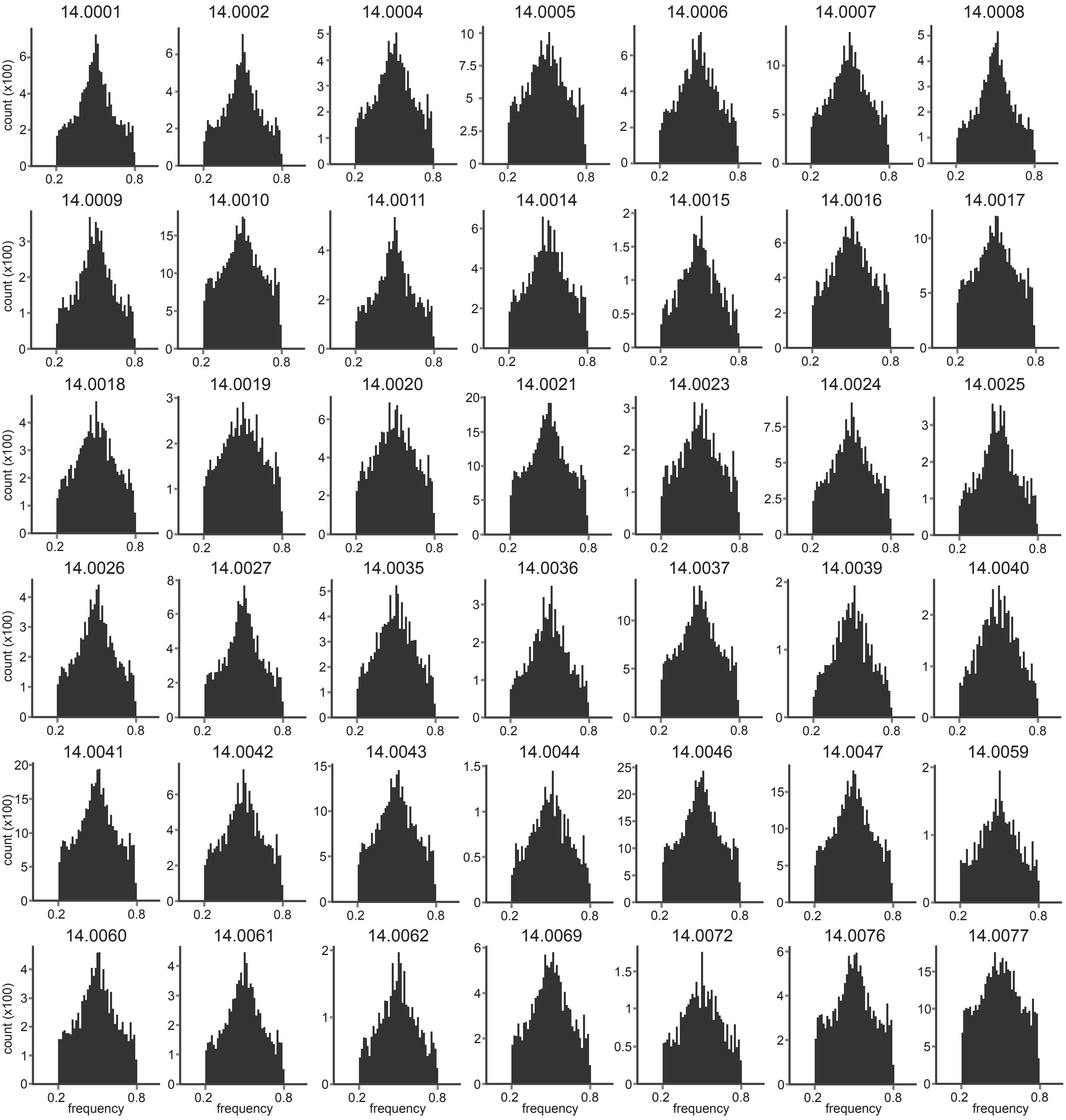

Figs S1: All 115 European *P. striiformis*-infected plant samples consist of a single genotype. Distribution of biallelic single nucleotide polymorphism read frequencies for all 115 P. striiformis field samples from 2014 (RNA-seq).

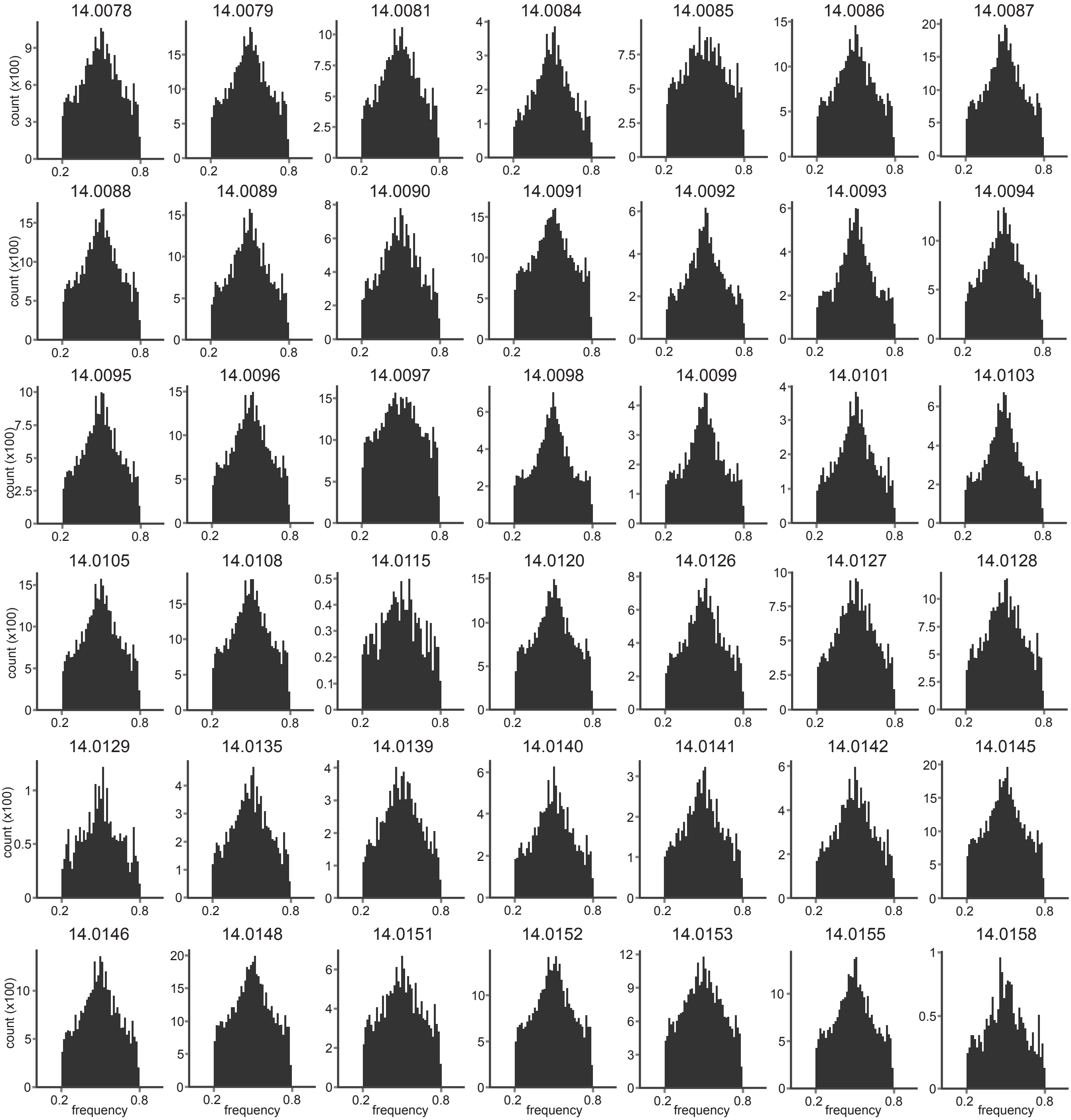

Figs S2: All 115 European *P. striiformis*-infected plant samples consist of a single genotype. Distribution of biallelic single nucleotide polymorphism read frequencies for all 115 P. striiformis field samples from 2014 (RNA-seq).

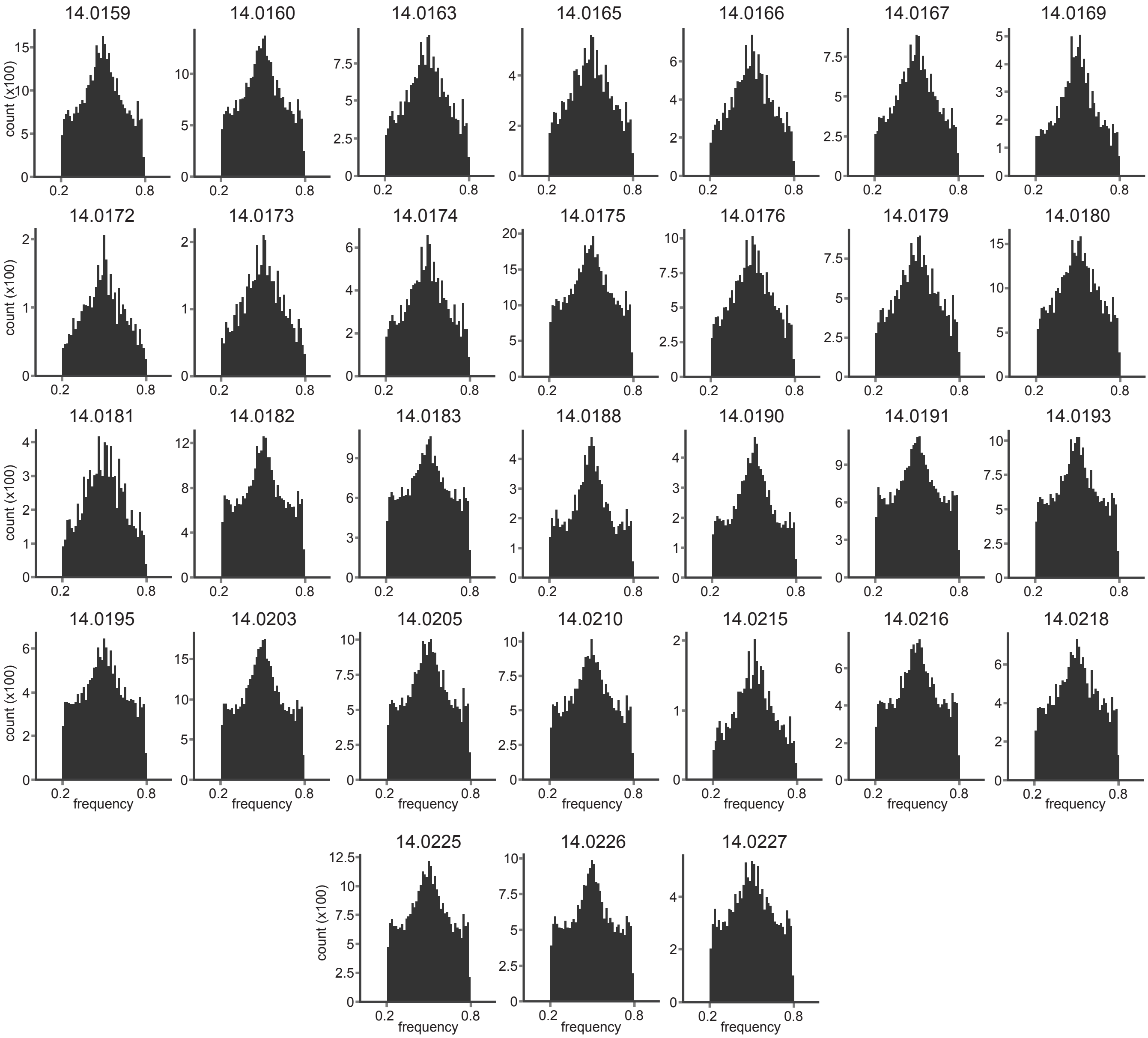

Figs S3: All 115 European *P. striiformis*-infected plant samples consist of a single genotype. Distribution of biallelic single nucleotide polymorphism read frequencies for all 115 P. striiformis field samples from 2014 (RNA-seq).

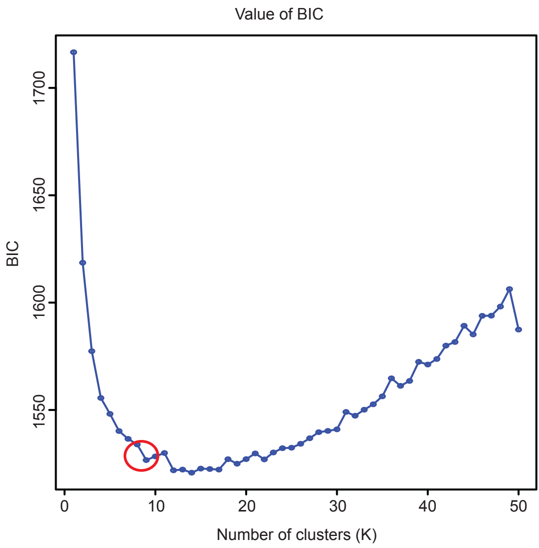

Fig S4: Multivariate discriminant analysis of principal components (DAPC). Bayesian information criterion (BIC) indicated eight to nine as the optimum clustering solution. The Y-axis corresponds to the BIC, a goodness-of-fit measurement calculated for each K. The elbow in the BIC values (K = 8-9) indicates the optimal number of population divisions.

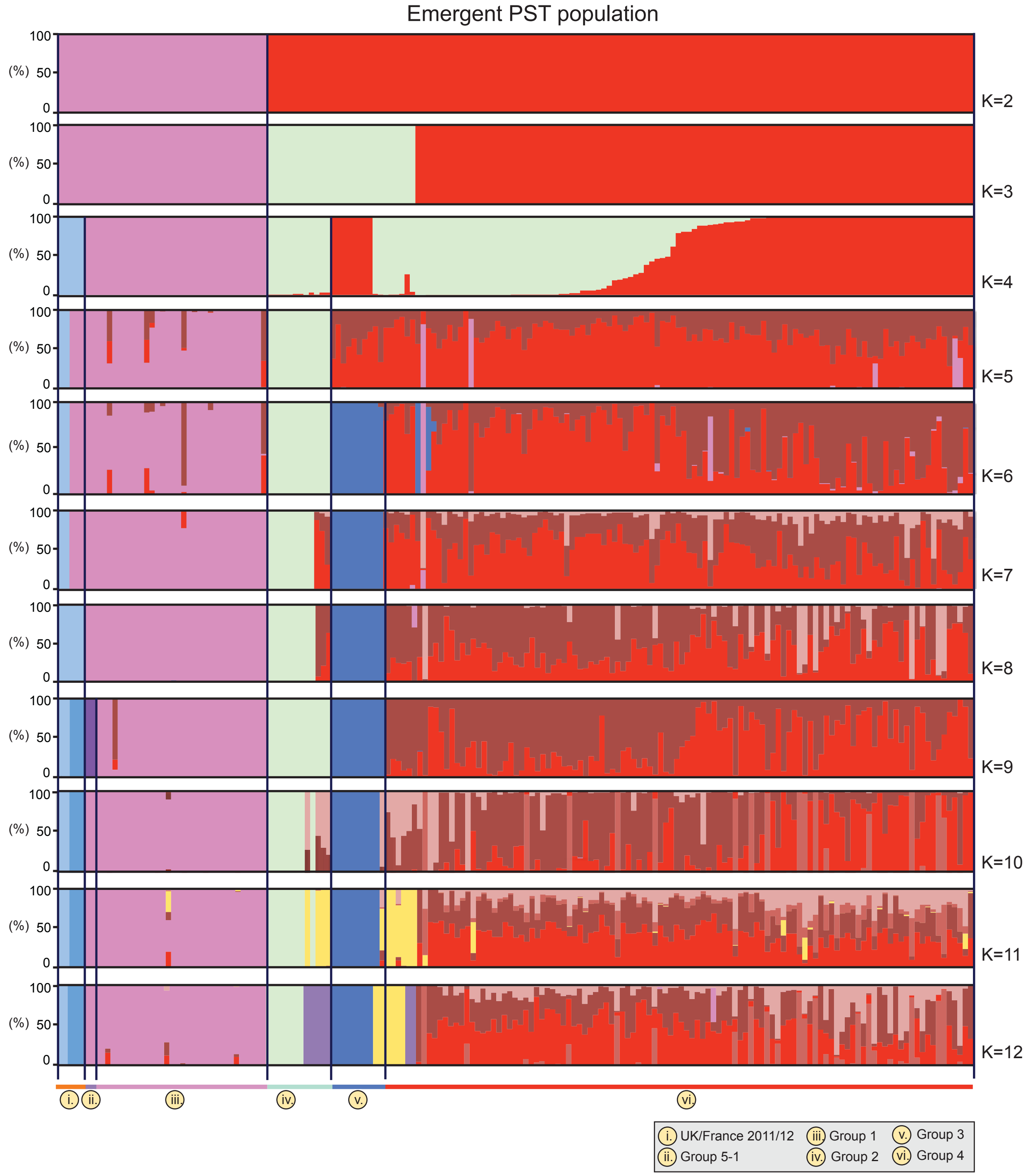

Fig S5: Multivariate discriminant analysis of principal components (DAPC) identified six genetic groups in the emergent *P. striiformis* population. DAPC analysis of 70,565 biallelic synonymous single nucleotide polymorphism (SNP) sites indicated stabilization of the emergent *P. striiformis* population at K = 8, with isolates assigned to six clearly definable homogeneous groups of individuals. Analysis was carried out for K = 2-12. Bars represent estimated membership fractions for each individual.

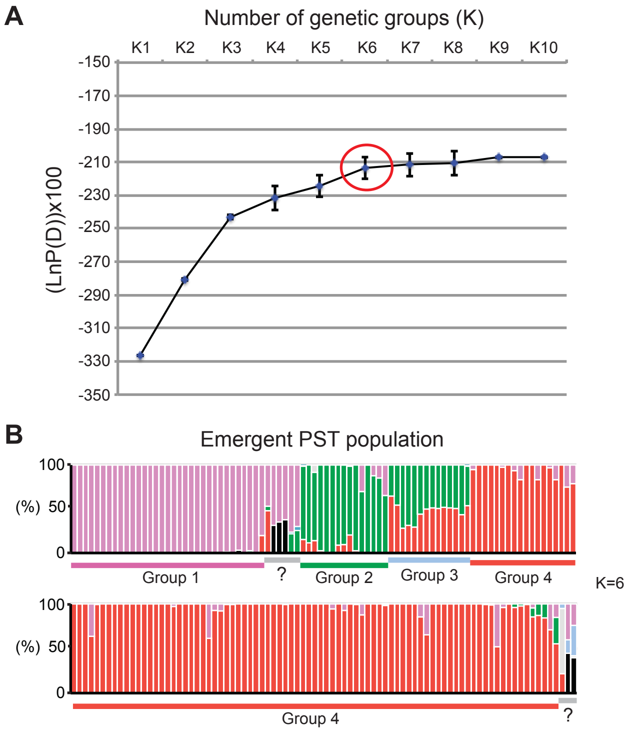

Fig S6: Distribution of the average log probability (LnP(D)) of each K value for the emergent *P. striiformis* population. (A) STRUCTURE analysis of 70,565 biallelic synonymous single nucleotide polymorphism (SNP) sites indicated stabilization of the emergent *P. striiformis* population at K = 6. **(B)** *P. striiformis* isolates were only clearly assigned to four of the six population clusters.

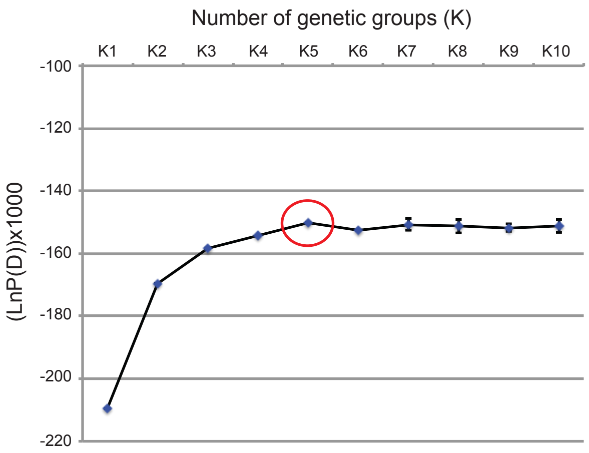

Fig S7: Distribution of the average log probability (LnP(D)) of each K value for the European *P. striiformis* population. STRUCTURE analysis indicates stabilization of the emergent *P. striiformis* population at K = 5.

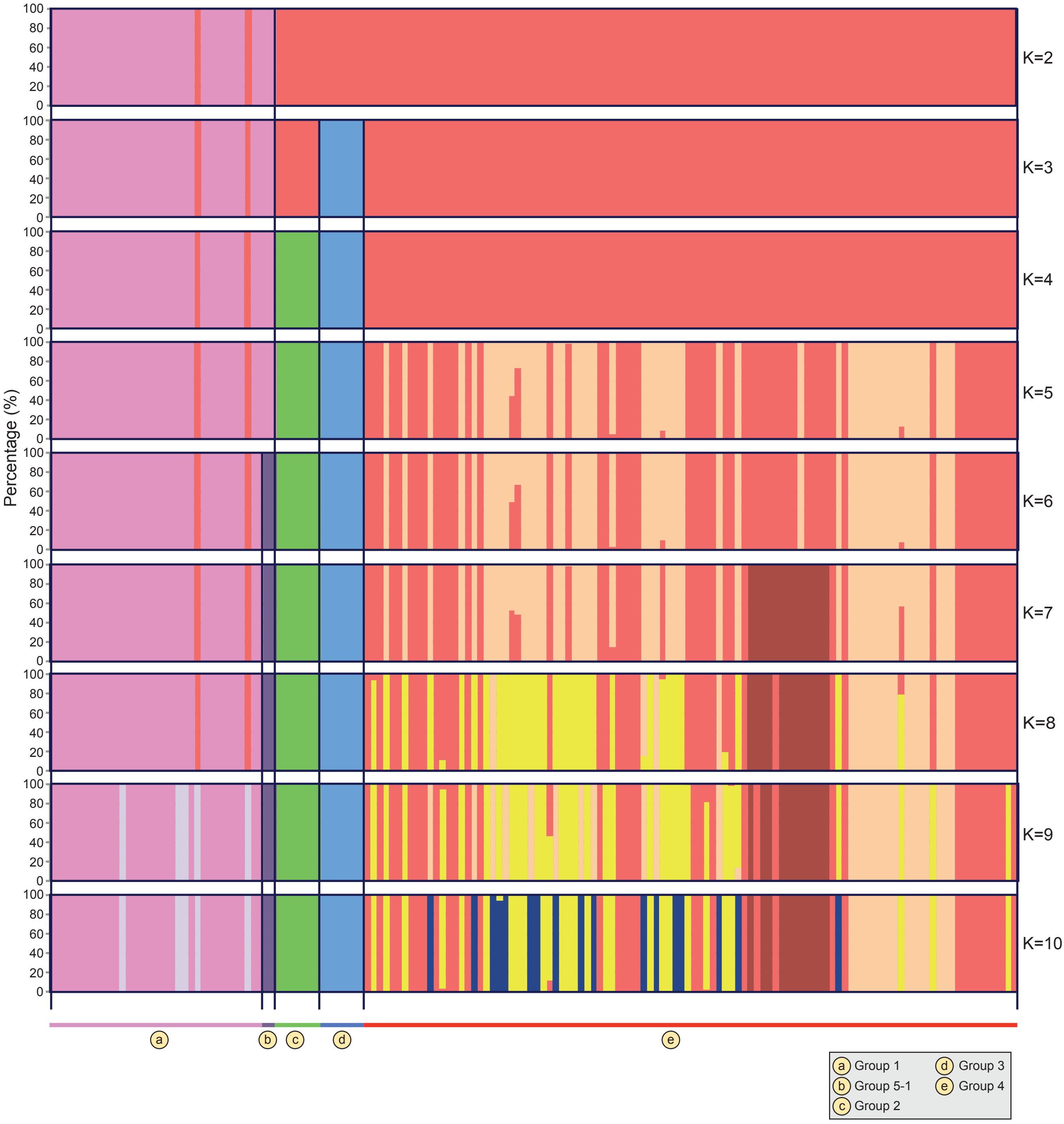

Fig S8: Multivariate discriminant analysis of principal components (DAPC) identified five genetic groups in the European *P. striiformis* population. DAPC analysis of 36,921 biallelic synonymous SNP sites indicated stabilization of the European population at K = 5. Analysis was carried out for K = 2-10. Bars represent estimated membership fractions for each individual.

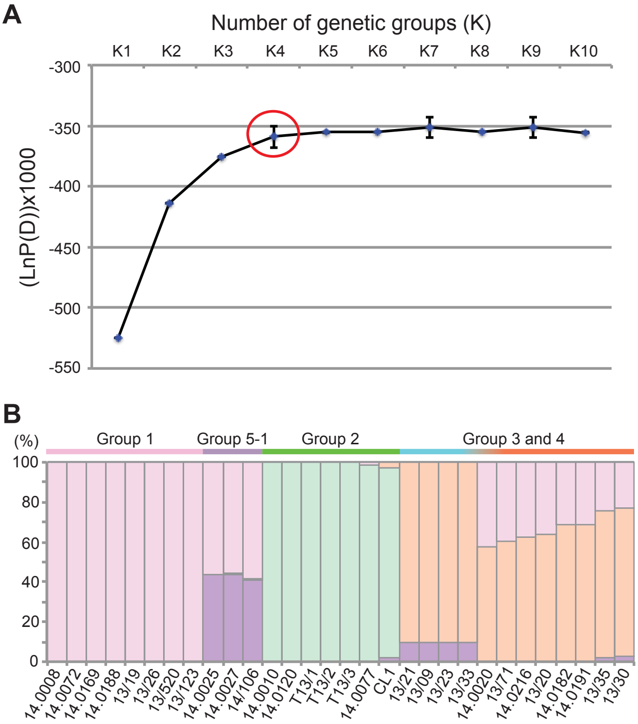

Fig S9: Assignment of the UK Kranich isolate (14/106) to genetic Group 5-1. (A) A total of 29 representative isolates were selected for analysis with *P. striiformis* 14/106, which represented an average of five isolates from each of the five genetic groups. STRUCTURE analysis was undertaken using 33,431 biallelic synonymous SNP sites. Analysis of the distribution of the average log probability (LnP(D)) of each K value showed stabilization at K = 4. **(B)** Bars illustrate estimated membership fractions for each individual following STRUCTURE analysis.

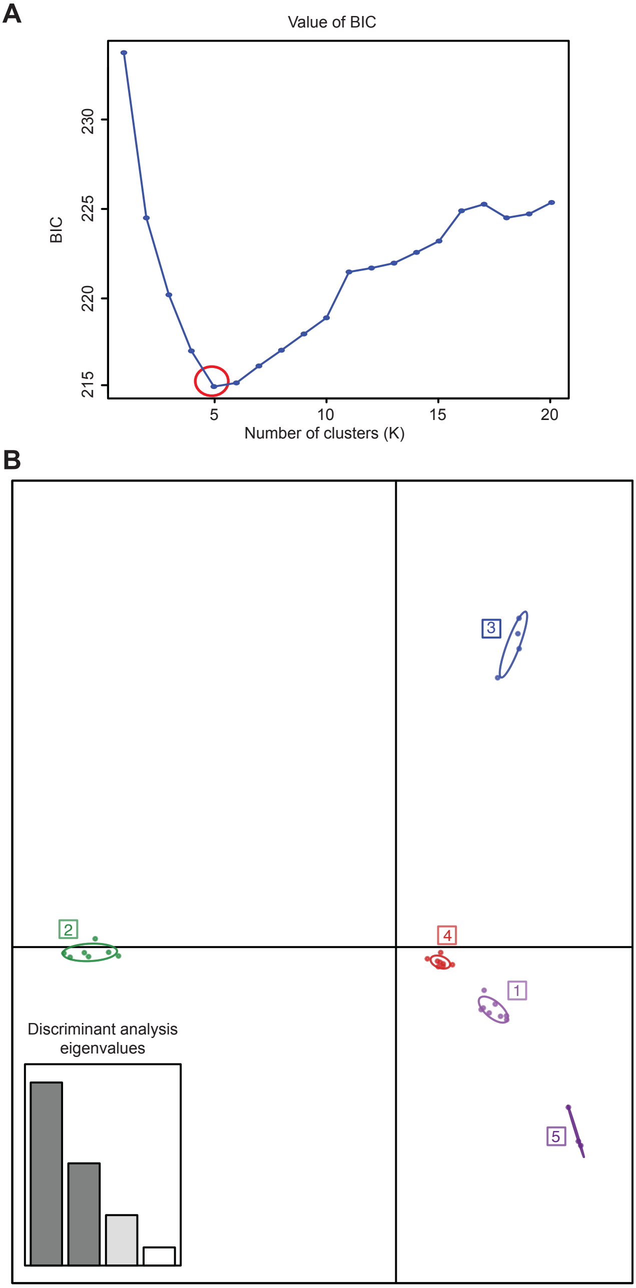

Fig S10: Multivariate discriminant analysis of principal components (DAPC) supported the assignment of the UK Kranich isolate to genetic Group 5-1. (A) The optimal predicted number of population clusters K for the dataset is five. The Y-axis corresponds to the Bayesian information criterion (BIC), a goodness-of-fit measurement calculated for each K. The elbow in the BIC values (K = 5) indicates the optimal number of populations. **(B)** Scatterplot using the first two principal components (Y-axis and X-axis, respectively) of DAPC analysis of 33,431 biallelic synonymous single nucleotide polymorphism (SNP) sites. Each symbol represents a single *P. striiformis* isolate, which is coloured according to assignment to one of five population clusters: Group 1 = pink, Group 2 = green, Group 3 = blue, Group 5-1 = purple, and Group 4 = red.

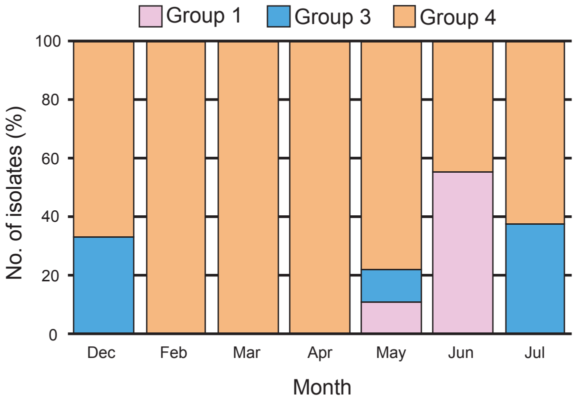

Fig S11: P. *striiformis* isolates captured in 2015 belonging to Group 1 also display seasonal specificity. *P. striiformis* isolates belonging to Group 1 were only identified in samples collected in May and June whereas isolates from Group 4 were identified throughout the growing season.

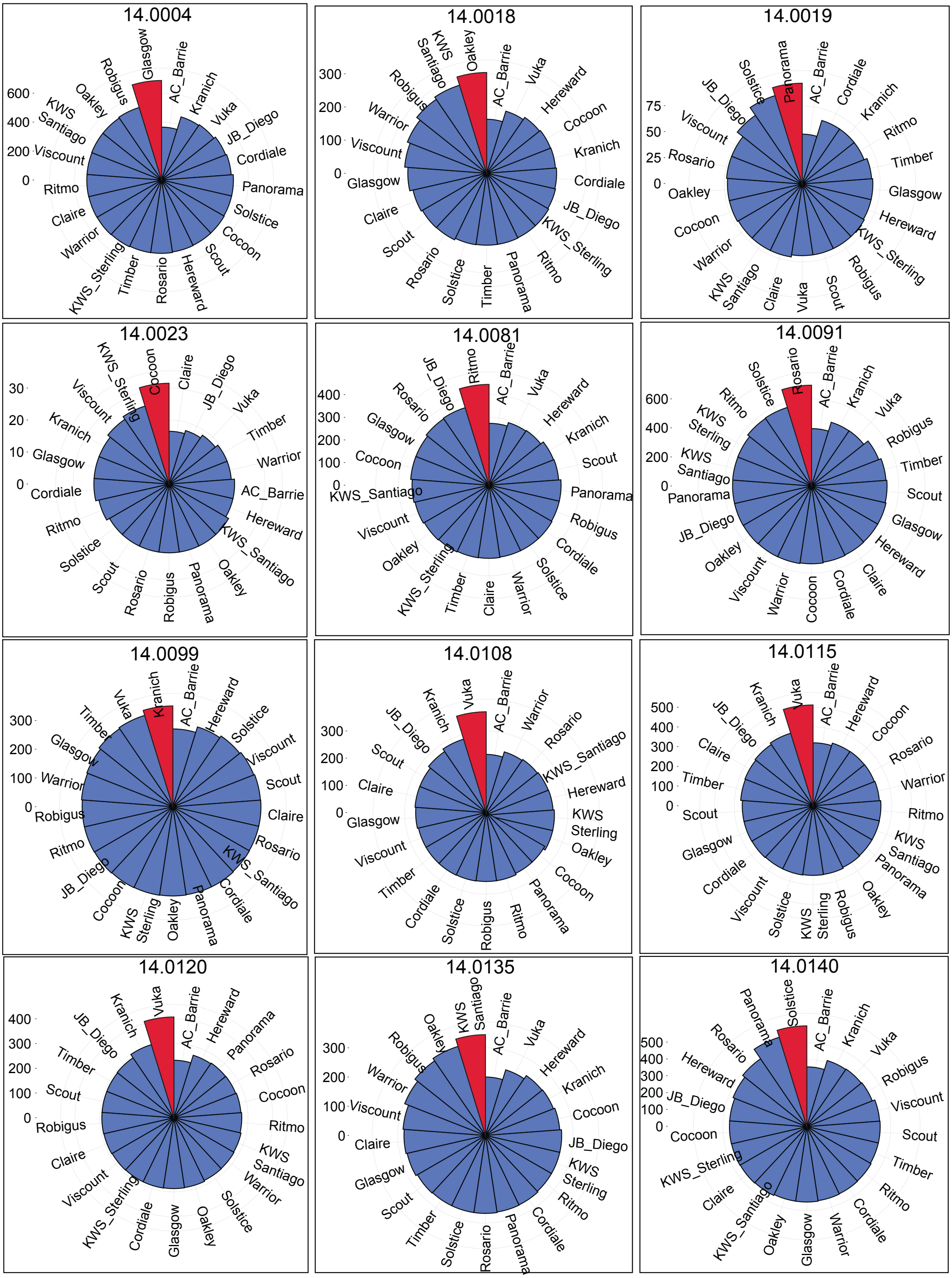

Figs S12: Identification of wheat varieties using transcriptome data generated directly from *P. striiformis*-infected field samples. A total of 21,505 SNP positions were used to differentiate wheat varieties, 1,831 of which were differential for the 21 wheat varieties assessed. For each SNP position, if the *P. striiformis*-infected field sample matched the sequence at a SNP site for a particular variety the position was scored as 1; if the site only partially matched the position was scored as 0.5; if the site had no match, the position was given a score of 0. The highest-scoring wheat variety is indicated by red shading in the corresponding segment.

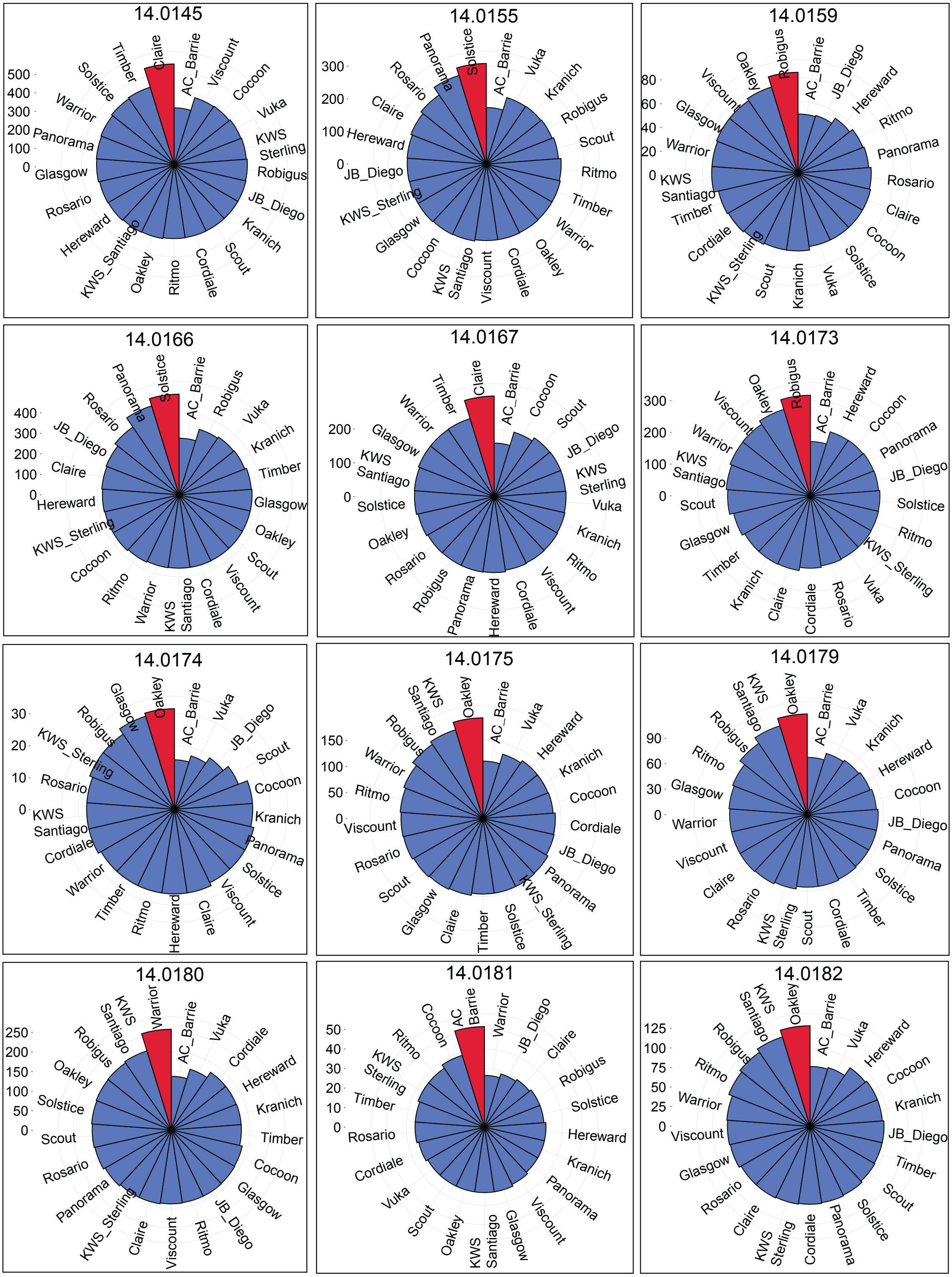

Figs S13: Identification of wheat varieties using transcriptome data generated directly from *P. striiformis*-infected field samples. A total of 21,505 SNP positions were used to differentiate wheat varieties, 1,831 of which were differential for the 21 wheat varieties assessed. For each SNP position, if the *P. striiformis*-infected field sample matched the sequence at a SNP site for a particular variety the position was scored as 1; if the site only partially matched the position was scored as 0.5; if the site had no match, the position was given a score of 0. The highest-scoring wheat variety is indicated by red shading in the corresponding segment.

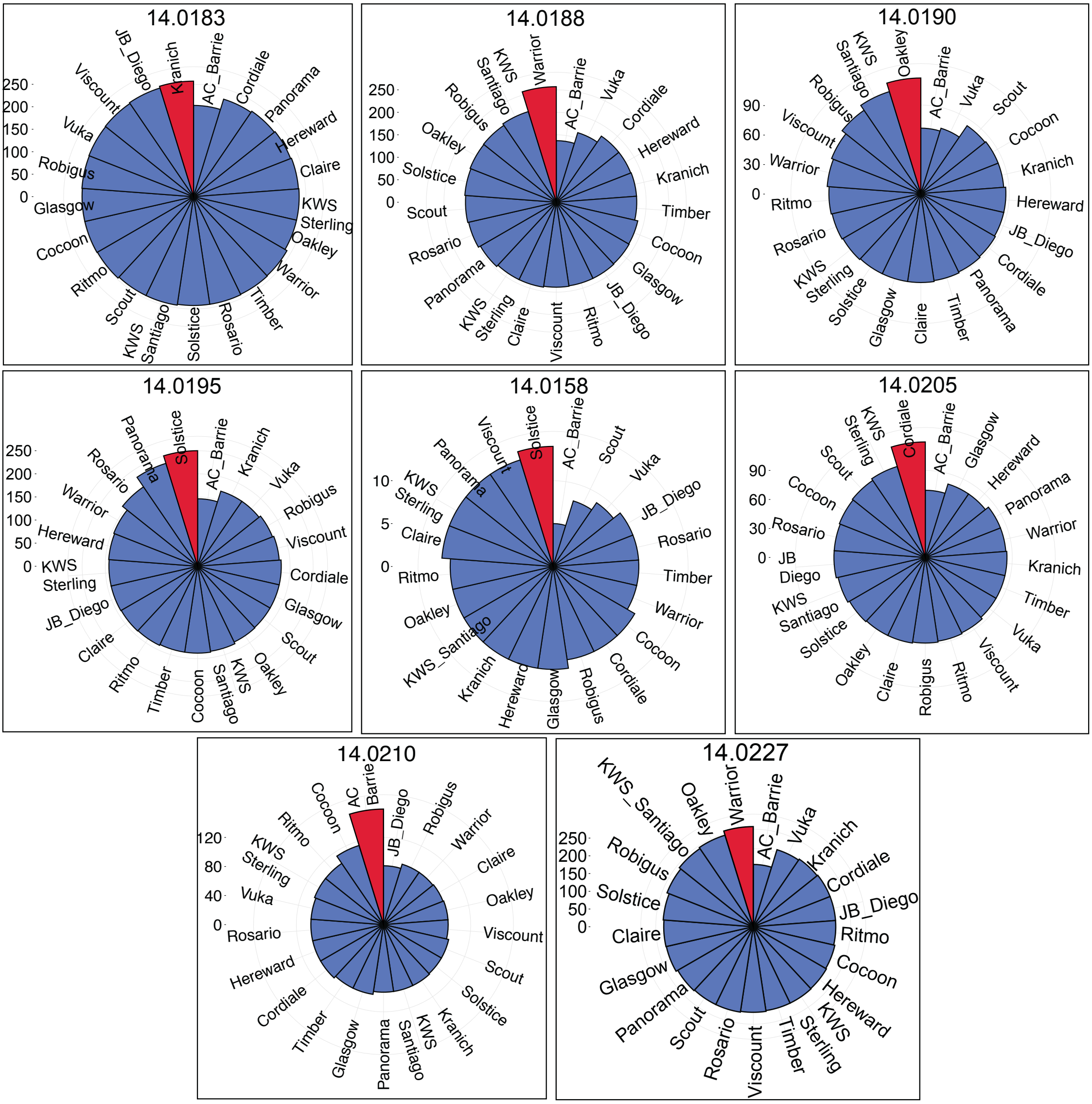

Figs S14: Identification of wheat varieties using transcriptome data generated directly from *P. striiformis*-infected field samples. A total of 21,505 SNP positions were used to differentiate wheat varieties, 1,831 of which were differential for the 21 wheat varieties assessed. For each SNP position, if the *P. striiformis*-infected field sample matched the sequence at a SNP site for a particular variety the position was scored as 1; if the site only partially matched the position was scored as 0.5; if the site had no match, the position was given a score of 0. The highest-scoring wheat variety is indicated by red shading in the corresponding segment.
